# Elevated ^18^F-FDG accumulation in a malignant epithelioid angiomyolipoma: a case report and review of literature

**DOI:** 10.3389/fonc.2025.1555092

**Published:** 2025-04-16

**Authors:** Li Zhang, Leqing Chen, Yinqian Deng, Huanyu Chen, Yujun Wu, Peng An, Jun Fan, Dawei Jiang, Xiaoli Lan, Wei Cao

**Affiliations:** ^1^ Department of Nuclear Medicine, Union Hospital, Tongji Medical College, Huazhong University of Science and Technology, Wuhan, China; ^2^ Hubei Province Key Laboratory of Molecular Imaging, Wuhan, China; ^3^ Department of Radiology, Union Hospital, Tongji Medical College, Huazhong University of Science and Technology, Wuhan, China; ^4^ Department of Radiology, Xiangyang No.1 People’s Hospital, Hubei University of Medicine, Xiangyang, China; ^5^ Department of Pathology, Union Hospital, Tongji Medical College, Huazhong University of Science and Technology, Wuhan, China

**Keywords:** epithelioid angiomyolipoma, 18 F-FDG PET/CT, tumor metabolism, cancer metabolism, tuberous sclerosis complex (TSC)

## Abstract

Epithelioid angiomyolipoma (EAML) is a tumor with malignant potential, as evidenced by its pathological features. Further investigation into its additional characteristics, particularly in imaging, is of great significance for non-invasive detection methods to understand its malignant potential. In this context, we present a case study of a 47-year-old male patient with a right renal EAML. The patient underwent nephrectomy but subsequently developed liver metastasis. Next-generation sequencing confirmed mutations of tuberous sclerosis 2 (TSC2) in both the primary and metastatic lesions. Consequently, the patient received maintenance treatment with the mTOR inhibitor, everolimus. However, treatment was discontinued after six months due to disease progression. Subsequent ^18^F-FDG PET/CT imaging revealed a large heterogeneous hypermetabolic mass in the liver, along with two other hypermetabolic metastases near the liver capsule. The patient’s prognosis was poor, with indicators such as TSC2 mutation, tumor necrosis, high Ki-67 expression, and α-SMA-negative fibroblasts. Despite reoperation, the patient still succumbed to disease progression. The occurrence of malignant metastatic EAML detected using ^18^F-FDG PET/CT imaging is infrequent. We conducted a comprehensive review of the relevant literature on ^18^F-FDG PET/CT imaging for EAML. Notably, this article emphasizes that elevated ^18^F-FDG uptake in EAML may serve as a novel indicator of malignant EAML.

## Introduction

Angiomyolipoma (AML) is a well-characterized mesenchymal neoplasm classified within a unique category of perivascular epithelioid cell tumors. It predominantly occurs in the kidneys, with the liver being the second most prevalent site of occurrence. Epithelioid angiomyolipoma (EAML) constitutes less than 5% of all AML cases (1). EAML have the potential to exhibit malignant behavior, with documented rates of metastasis or recurrence ranging from 5% to 50% (2–4). Distinguishing EAML from hepatocellular carcinoma or renal cell carcinoma can be challenging during imaging assessments. Immunohistochemistry (IHC) plays a crucial role in diagnosis, with positive staining for melanocytic markers, such as HMB-45, and muscle markers, such as α-smooth muscle actin (α-SMA), being the most significant features (1).

Positron emission tomography/computed tomography (PET/CT) is a widely utilized imaging modality for staging tumors, predicting malignancy, monitoring treatment response, and assessing prognosis (5, 6). Nonetheless, its application in EAML has rarely been reported, and the metabolic pathways of ^18^F-FDG in EAML have/ been infrequently explored. In this study, we report a case of hepatic-metastatic EAML that exhibited increased ^18^F-FDG uptake on PET/CT. Furthermore, we conducted a comprehensive literature review on EAML cases assessed with ^18^F-FDG PET/CT to evaluate its diagnostic value.

## Case presentation

**Figure 1:**
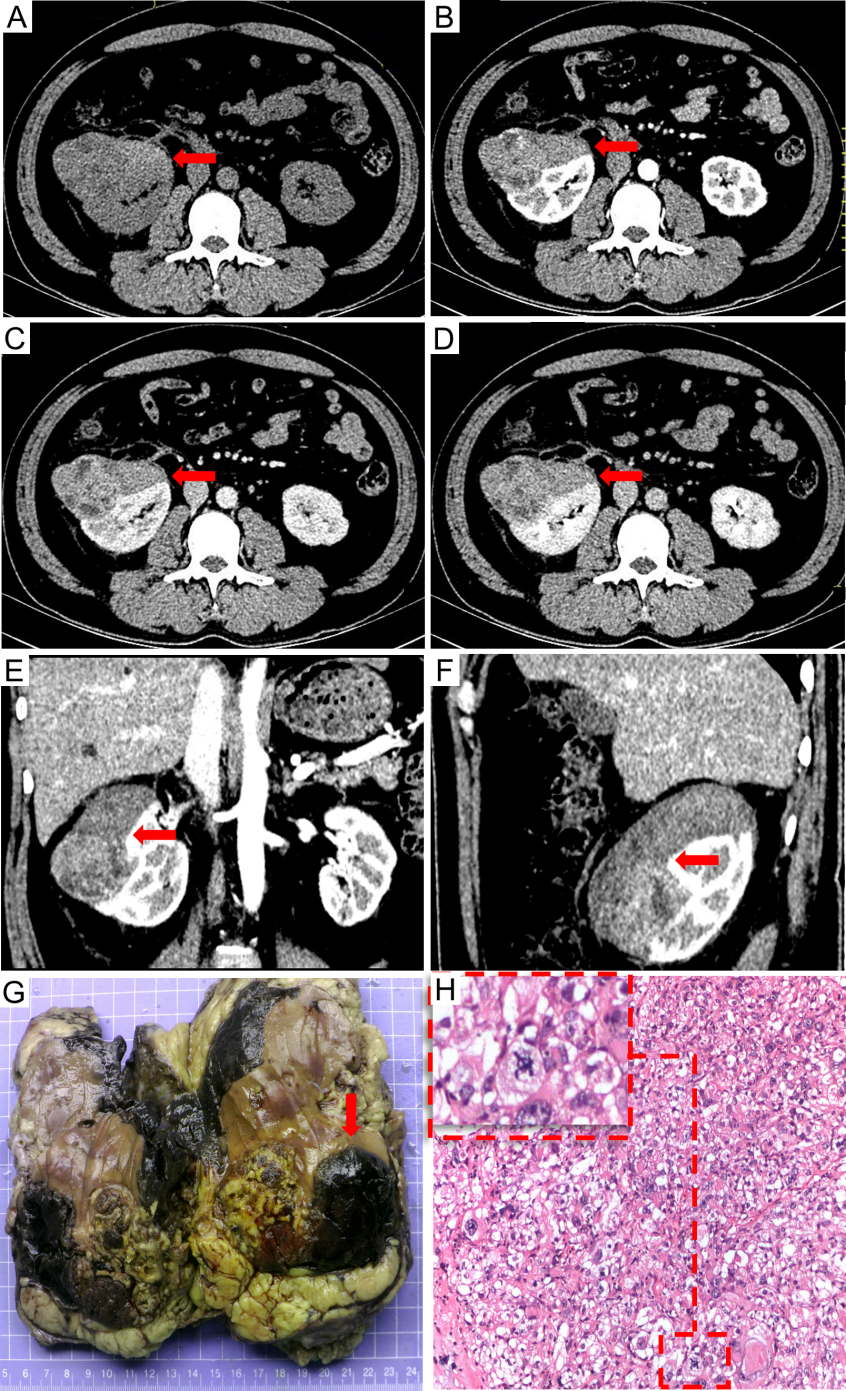
The preoperative contrast-enhanced CT scan: **(A)** non-contrast, **(B)** arterial phase, **(C)** venous phase, **(D)** delayed phase, **(E)** sagittal planes in arterial phase, and **(F)** coronal planes in arterial phase. Contrast-enhanced CT scan revealed a neoplastic lesion in the right kidney measuring 9.9 cm × 7.6 cm × 6.2 cm size neoplastic lesion in the right renal with heterogeneous enhancement in the arterial phase and wash out in the delayed phase. Multiple tumoral necrosis were suspected. The pathological diagnosis identified the tumor as epithelioid angiomyolipoma. The tumor exhibited an invasive growth pattern, enveloping and fusing with the right kidney. Macroscopic appearances revealed hemorrhage and necrosis [**(G)** the red arrow]; Additionally, significant pathological mitotic figures were observed within the tumor cells [**(H)** H&E stain, 100×, the red frame].

**Figure 2:**
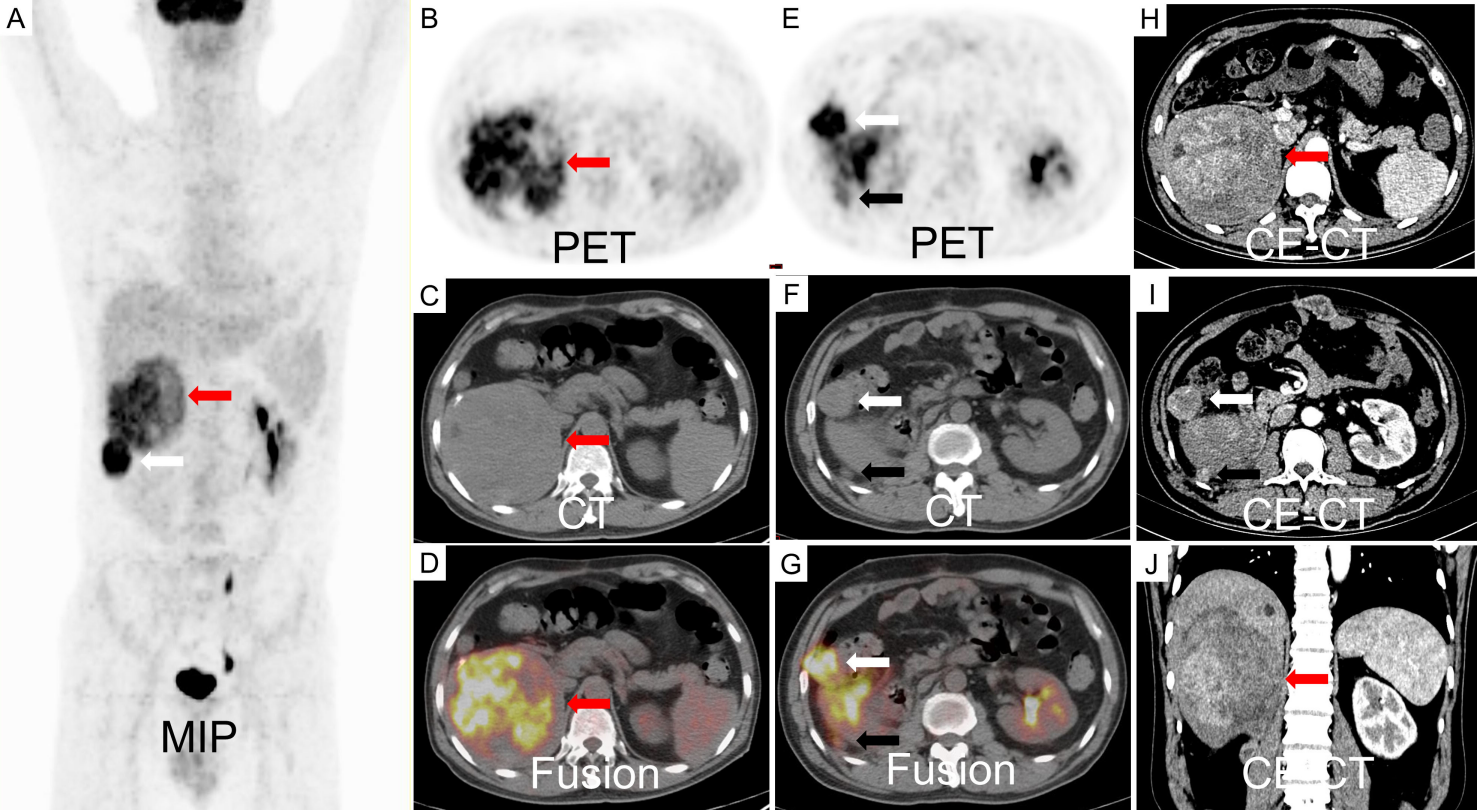
An ^18^F-FDG PET/CT scan revealed a large, inhomogeneous hypermetabolic mass in the liver, characterized by a SUVmax of 8.8, as indicated by the red arrow. Additionally, two hypermetabolic metastases were identified adjacent to the liver capsule, with SUVmax values of 12.1 (white arrow) and 3.1 (black arrow), respectively **(A-G)**. The contrast-enhanced CT scan demonstrated inhomogeneous enhancement of the metastases during the arterial phase **(H-J)**.

A 47-year-old male patient presented with one-week history of hematuria and lumbar discomfort. An abdominal contrast-enhanced computed tomography (CE-CT) scan identified a neoplastic lesion measuring 9.9 cm × 7.6 cm × 6.2 cm in the right kidney, without macroscopic lymphadenopathy or distant metastasis (**Figure 1**). The lesion demonstrated heterogeneous enhancement during the arterial phase and wash-out in the delayed phase, suggesting the possibility of renal carcinoma or angiomyolipoma with tumoral necrosis. Subsequently, a right nephrectomy was performed, and histopathological analysis confirmed the diagnosis of EAML with tumoral necrosis (**Figure 1**). Immunohistochemical analysis demonstrated positive expression of HMB45, Melan-A, TFE3, and P53, whereas α-SMA and S-100 were negative. The Ki-67 labeling index was 40%. Nine months later, the patient felt upper abdominal pain, and MRI indicated liver metastasis measuring 6.5 cm × 5.0 cm. Subsequently, one month later, a partial hepatectomy was performed due to liver metastasis, which displayed a high risk of recurrence and progression upon pathological examination. Histopathological analysis revealed epithelioid tumor cells with marked heteromorphism, pathological mitosis, and tumor necrosis. The Ki-67 labeling index was 60%. Next-generation sequencing (NGS) of EAML revealed mutations in TSC2, TP53, and ATRX in resected lesions. Subsequently, the patient was administered everolimus at a dose of 10 mg/day. After a two-month treatment period, the tumor recurred at the right margin of the liver and exhibited progressive growth. The patient had no obvious adverse reaction. After four months, treatment was discontinued owing to tumor progression. To assess potential metastasis in the whole body, ^18^F-FDG PET/CT and CE-CT were performed. The ^18^F-FDG PET/CT scan revealed a huge heterogeneous hypermetabolic mass measuring 11.3 cm × 10.5 cm at the right margin of the liver, with a maximum standardized uptake value (SUVmax) of 10.7. Additionally, two lesions were detected at the anterior and posterior edges of the liver, measuring 3.2 cm × 2.9 cm and 0.9 cm × 0.8 cm, with SUVmax of 12.1 and 7.6, respectively. CE-CT revealed three prominent metastases in the arterial phase (**Figure 2**). Consequently, a surgical resection was performed. Regrettably, EAML recurred in the liver two months later, and the patient died soon after the rapid progression of the disease.

## Discussion

**Figure 3:**
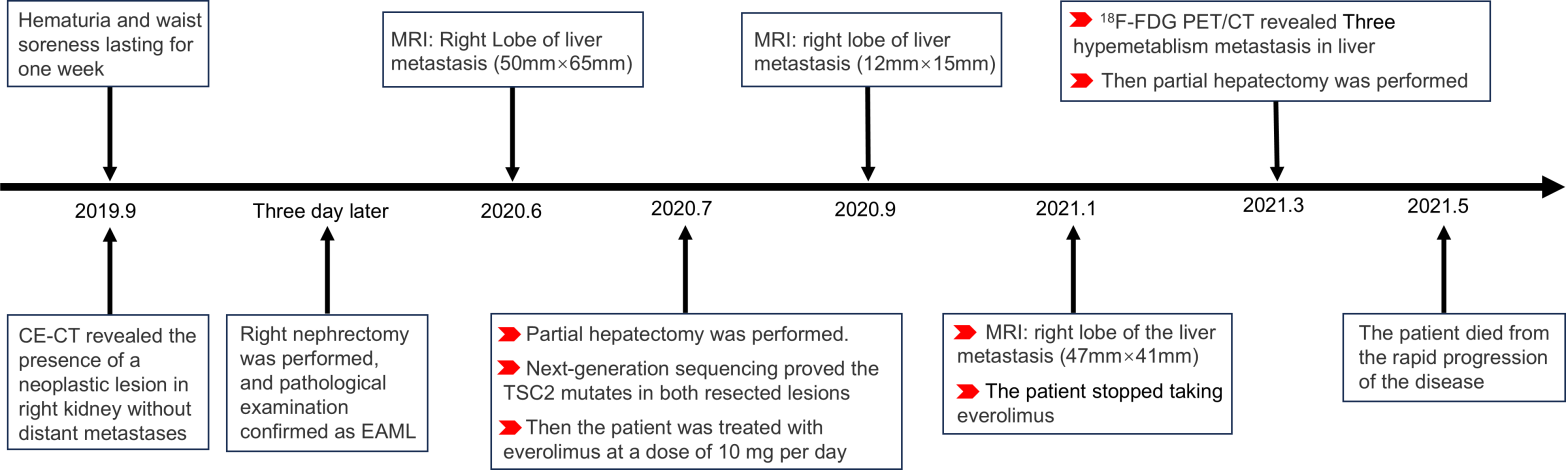
Flowchart: the detail of diagnosis and treatment in the patient.

EAML, a rare AML variant, is a potentially malignant neoplasm. The patient survived 32 months after the primary lesion was detected. The details of the diagnosis and treatment are shown in the flowchart (**Figure 3**). Relevant knowledge of this disease needs to be introduced before discussion. AML is a prevalent benign tumor with diverse cellular origins. Approximately 80% of AML cases are sporadic and frequently manifest as multiple or bilateral in individuals with tuberous sclerosis complex (TSC) (4). AMLs < 4 cm are usually asymptomatic and are often detected during imaging examinations after an accident. Symptomatic AMLs such as abdominal pain and distension are more prone to hemorrhagic shock (7). AML can be classified into classic and epithelial types. Different from classic AML, epithelioid cells in epithelioid AML account for at least 80% of tumors (4). Furthermore, approximately 27% of patients with EAML have concomitant TSC, whereas it appears in 6.7% of patients with classic AML. The average reported patient age for EAML is approximately ten years earlier than that for AML, possibly due to the easier onset of symptoms such as abdominal symptoms, hemorrhagic shock, and TSC in EAML (2, 3, 8).

The classic AML contains blood vessels, smooth muscle, and mature adipose tissue, whereas EAML often lacks mature adipose tissue. EAML cells are epithelioid and may be misdiagnosed as leiomyosarcoma, liposarcoma, and even carcinoma. Immunohistochemical staining revealed the expression of the muscle marker α-SMA and melanocytic markers HMB-45 and Melan-A (2), but was negative for markers of epithelial or neural cells. Treatment with everolimus has been found to be more effective in AML patients with TSC than in those without TSC (8). The potential malignant behaviors of renal EAML are associated with TSC or concurrent AML, necrosis, extrarenal extension and/or renal vein involvement, tumor size >7 cm, and carcinoma-like growth pattern (3). Brimo et al. (9) summarized four pathological malignant characteristics of renal EAML: 1. atypical epithelioid cells ≥ 70%, 2. ≥2 mitotic figures per 10 HPF, 3. atypical mitotic figures, and 4. tumor necrosis. The presence of three or more features was highly suggestive of malignant behavior.

Generally, EAML are detected using CT and MRI. Most EAMLs have a “fast-in and slow-out” pattern on CE-CT, which usually shows hyperdensity with or without an adipose component (11). On MRI, EAMLs are mainly isointense on T1WI, hypointense on T2WI, restricted diffusion on DWI, round tumor-kidney interface, and reticular (12). Classic AML generally has dysmorphic blood vessels and adipose tissues. Tumor adipose tissue, hemorrhage, and cystic degeneration are all sensitive to detection using CT and MRI (12, 13). Radiologically, EAML resembles hepatocellular carcinoma and renal cell carcinoma due to the scarcity of adipose tissue. Consequently, the uncertainty of imaging diagnosis and lack of consensus on diagnostic characteristics restrict the comprehension of EAML.

^18^F-FDG PET/CT is an examination to identify systemic lesions and assess the malignant degree of tumors by the “Warburg effect”. Reports on ^18^F-FDG PET/CT imaging of EAML are scarce. Several studies have characterized classic AMLs as non-hypermetabolic tumors. Lin et al. (14) reviewed 21 patients with renal AML who underwent PET and PET/CT imaging. The result demonstrated None of the 21 classic AMLs showed an SUVmax greater than 1.98. The result was also proved by Jiang et al. (15) for eight AMLs, in which none of the eight AMLs demonstrated SUVmax higher than 2.3. In the present case, ^18^F-FDG PET/CT was performed to detect metastatic lesions in patients with EAML. Three metastatic lesions in the liver were found to have increased ^18^F-FDG uptake, which has rarely been reported. Consequently, this finding prompted further investigation of the relationship between glucose metabolism and EAML.

Based on the published reports, the metabolism and clinicopathological features of EAML are summarized in **Table 1**. The results of our research showed that the rate of high ^18^F-FDG uptake in metastatic EMALs was 100% (6/6), while it was 60% (3/5) in primary EAMLs. All EAMLs exhibiting necrosis (4/4) showed hypermetabolic lesions. Among the eight patients with hypermetabolic lesions, four (50%) were proven to have metastasized in follow-up studies. Consequently, SUVmax may serve as a potential marker for predicting the malignant potential of EAML, particularly in metastatic lesions. Two patients presented with both hypermetabolic and non-hypermetabolic lesions. The discrepancy between the primary lesions and metastatic lesions of ^18^F-FDG metabolism probably showed heterogeneity between the primary lesion and metastases. Consequently, more attention should be paid to distinguishing EAML metastases from other tumors. In addition to the above summary, the study also indicated that ^18^F-FDG PET/CT could monitor everolimus response assessment (16) and distinguish vascular invasion from thrombus in AML after nephrectomy from the uptake of ^18^F-FDG by the tumor (17).

**Table 1:** Cases of EAML with ^18^F-FDG PET/CT examination.

Author	Gender	Age	Type	Location	Size (cm)	α-SMA	Necrosis	SUVmax	Recurrence/metastasis
Our case	Male	47	Renal EAML	Liver metastasis	11.3	–	+	10.7	+
					3.3			12.1	
					1.4			3.8	
Vicens (22)	Female	62	Renal EAML	Liver metastasis	10.5	–	+	7.9	+
Dong (23)	female	21	Renal EAML	Renal primary	2.8	Unknow	Unknow	4.4	–
Andrew (17)	Female	52	Renal EAML	Vein metastases	Unknow	Unknow	Unknow	12	+
Galatola (24)	Male	36	Renal EAML	Renal primary	30	+	Unknow	Not elevated	–
Zhang (25)	Female	42	HEAML	Liver	4.0	+	–	8.8	Unknow
					2.0	+	–	Not elevated	
Wang (26)	Female	50	HEAML	Liver primary	8.4	Unknow	+	8.8	–
Marcuzzi (27)	Female	47	HEAML	Liver primary	<12	+	–	Not elevated	+
				Renal metastasis	<6.2		+	14.3	
Anwar (16)	Female	27	Adrenal EAML	Renal primary	9	Unknow	Unknow	5.9	–

AML, angiomyolipoma; EAML, epithelioid AML; HEAML, hepatic EAML.

The uptake of ^18^F-FDG is mainly determined by glucose transport, tumor blood flow, and glycolytic rate. AML is regarded as a benign hypervascular tumor with the potential for spontaneous hemorrhage. Consequently, blood flow cannot explain the low uptake of ^18^F-FDG in AML (14). Glucose transporter-1 (GLUT1) is one of the main factors that affect glucose transport. However, Zhang et al. (18) tested the expression of GLUT1 in seven cases of EAML and found that none of the EAML lesions expressed GLUT1. Therefore, glucose transport may not be the primary factor responsible for the uptake of ^18^F-FDG in EAML patients. The higher frequency of reports in hypermetabolic EAML proves that alternative mechanisms facilitate glucose uptake in EAML. TSC mutations occur more frequently in EAML than AML. The loss of 1/2 function in TSC leads to constitutive mTORC1 activation, which promotes cellular metabolism, including glycolytic rate (16, 19), and is associated with elevated ^18^F-FDG uptake in PET/CT scans.

Tumor metabolism is associated with various pathological indicators. Ki-67 labeling indicates that the cell proliferation rate, with high expression in Ki-67 labeling, is correlated with poorer prognosis and higher ^18^F-FDG uptake (10, 20), which is consistent with our report. Furthermore, α-SMA is the main indicator of Cancer-Associated Fibroblasts (CAFs). High expression of α-SMA in fibroblasts in oral squamous cell carcinomas has been shown to be associated with a poorer prognosis. Conversely, Anwaier et al. showed that α-SMA-negative fibroblasts were significantly correlated with higher recurrence and metastasis rates in immunohistochemical indexes of fifty-seven patients with renal EAML (21). This finding suggests that the metastatic mechanism of EAML may differ from that of other tumors. In the present case, the patient underwent a right nephrectomy, followed by recurrent liver metastasis and progression. Rapid progression of the disease may have been influenced by the presence of α-SMA-negative fibroblasts, elevated Ki-67 labeling, increased ^18^F-FDG uptake, and necrosis.

## Conclusion

We identified a rare case of renal EAML with liver metastasis that demonstrated high ^18^F-FDG uptake on PET/CT. Then, We comprehensively reviewed case reports on hypermetabolic EAML, analyzing the causes of ^18^F-FDG uptake and its association with malignancy, The reason for ^18^F-FDG uptake in EAML may be associated with TSC mutation and high Ki-67 expression. This study demonstrated the high sensitivity of PET/CT in detecting metastatic lesions of EAML. The uptake of ^18^F-FDG by EAML may be related to an increased glycolytic rate resulting from TSC mutations. Furthermore, the malignant potential of EAML is influenced by the expression of α-SMA and Ki-67, and the presence of necrosis within the tumor.

## Data Availability

The original contributions presented in the study are included in the article/supplementary material. Further inquiries can be directed to the corresponding author.
